# Immune dysregulation and pathogen prevalence in Mycoplasma pneumoniae pneumonia: a retrospective insight in children

**DOI:** 10.3389/fped.2026.1858160

**Published:** 2026-08-21

**Authors:** Yuzuo Geng, Jiang Yang, Yuanyuan Wang, Guiling Wang, Qiaowen Xu, Wenmiao Zhong, Xianzhi Ren

**Affiliations:** 1 Department of Pediatric, Suqian Affiliated Hospital of Nanjing University of Traditional Chinese Medicine, Suqian Hospital of Traditional Chinese Medicine, Suqian, Jiangsu, China; 2 The First Clinical Hospital of Nanjing University of Traditional Chinese Medicine, Nanjing, Jiangsu, China; 3 Department of Traditional Chinese Medicine, Nanjing Children’s Hospital, Nanjing, Jiangsu, China; 4 Department of Pediatric, Nantong Hospital of Traditional Chinese Medicine, Nantong, Jiangsu, China

**Keywords:** children, lymphocyte subsets, Mycoplasma pneumoniae pneumonia, pathogenic characteristics, pleural effusion

## Abstract

**Objective:**

To explore the etiological characteristics and lymphocyte subtype features of pediatric mycoplasma pneumonia complicated with pleural effusion, clarify the influencing factors and predictive indicators of its occurrence, and analyze the manifestations of immune dysregulation in the children.

**Methods:**

This study retrospectively selected 104 children diagnosed with mycoplasma pneumonia in the pediatric department of our hospital from February 2023 to February 2025. The age range was 1–13 years old. They were divided into the mycoplasma pneumonia with pleural effusion group (46 cases) and the non-pleural effusion group (58 cases) based on chest ultrasound examination. Pathogenic identification was performed by matrix-assisted laser desorption/ionisation time-of-flight mass spectrometry, and qRT-PCR. Peripheral blood lymphocyte subsets (CD3⁺T, CD4⁺T, CD8⁺T, CD19⁺B, CD56⁺NK) were analyzed by flow cytometry. Risk factors were identified using multivariate logistic regression, and their predictive performance was evaluated using receiver operating characteristic (ROC) curve analysis.

**Results:**

Among the 104 children with Mycoplasma pneumoniae, the incidence of pleural effusion was 44.23% (46/104). A total of 59 pathogens were detected in the respiratory specimens of the aforementioned children. Among them, viruses accounted for the highest proportion (44.07%), followed by Mycoplasma pneumoniae (32.20%) and bacteria (23.73%). Mixed infections accounted for 66.10%, while single infections accounted for 33.90%. Compared with the non-effusion group, the levels of CD3 ^+^ T, CD4 ^+^ T, and CD8 ^+^ T were reduced in the complicated group, while the levels of CD19 ^+^B and CD56 ^+^NK were significantly increased (*P* < 0.05). α1-AG, CRP, and GAG were independent risk factors for pleural effusion, with OR values of 2.277 (95% CI: 1.365–3.800), 1.982 (95% CI: 1.245–3.155), and 2.502 (95% CI: 1.480–4.229), respectively (*P* < 0.05). The combined detection of α1-AG, CRP, and GAG achieved an AUC of 0.924, with sensitivity of 89.13% and specificity of 87.93%, significantly outperforming any single marker.

**Conclusion:**

Among the 104 children diagnosed with mycoplasma pneumonia, viral pathogens were the most frequently detected in the respiratory tract, and the proportion of mixed infections was relatively high. Children with pleural effusion showed more significant immune dysfunction. The combined prediction model of these three indicators has a high diagnostic value (AUC = 0.924), providing a reference for early clinical warning.

## 1 Introduction

Children possess an immature immune system and an underdeveloped respiratory mucosal barrier, rendering them highly susceptible to lower respiratory tract infections. Community-acquired pneumonia (CAP) is one of the most frequent and severe respiratory illnesses in paediatric populations (1). Mycoplasma pneumoniae pneumonia (MPP) is a well-established leading pathogen of lower respiratory tract infection and a primary cause of CAP in children, responsible for 10%–40% of cases. In recent years, periodic outbreaks have been reported in several regions, creating persistent challenges for paediatric diagnosis and management (2). Clinical practice shows that most children with mycoplasma pneumonia have a good prognosis after receiving standardized anti-infection treatment. However, some cases progress rapidly and develop into severe pneumonia, accompanied by various pulmonary complications. Among them, pleural effusion is the most common, and also includes lung abscess, atelectasis, etc. These complications would directly aggravate the pathological damage to the lungs, disrupt the pulmonary ventilation and gas exchange functions, and significantly affect the prognosis and recovery of the children (3, 4). Relevant clinical research data show that the incidence of pleural effusion in children with mycoplasma pneumonia is approximately 10%–15% (5, 6). The occurrence of pleural effusion will aggravate respiratory symptoms such as coughing and wheezing. Some children may present with breathing difficulties and chest tightness, and the hospitalization period is often prolonged, with increased medical resource consumption, and it may also have a long-term impact on lung function. However, pleural effusion can be seen in children with mycoplasma pneumonia of different severity levels, not only in severe cases. Its occurrence is related to multiple factors and should not be directly used as the sole basis for judging the severity of the disease (6).

Children with MPP and pleural effusion typically present with fever and cough. However, clinical manifestations vary widely due to differences in immune status, infection severity, and pathogen virulence, making early recognition and diagnosis difficult (7). Therefore, clarifying the pathogenic profile is essential for guiding targeted treatment and improving therapeutic efficacy. Pleural effusions caused by different pathogens differ in biochemical properties, inflammatory markers, and treatment responses. Accurate pathogenic identification facilitates differential diagnosis from bacterial empyema, tuberculous pleurisy, and other conditions, thereby avoiding inappropriate empirical therapy, progressive complications, and unnecessary medical resource consumption. Furthermore, *M. pneumoniae* infection triggers intense systemic inflammation and aberrant immune activation, leading to coagulation disorders and immune dysfunction. These disturbances disrupt pleural capillary permeability and directly contribute to pleural fluid formation, prolonging the clinical course and potentially impairing lung development (8).

Immune dysregulation plays a central role in the pathogenesis of infectious diseases. Lymphocyte subsets are key cellular components of the host immune response, and quantitative and functional changes directly reflect immune status (9). CD3⁺ T cells represent the total T cell pool, and their decline indicates the suppression of overall cellular immune function; CD4⁺ T helper cells coordinate the anti-mycoplasma immune response, and their reduction might weaken the Th1 type protective response; CD8⁺ cytotoxic T cells are responsible for eliminating infected cells, and their decreased levels are associated with the delayed clearance of intracellular pathogens such as viruses (10); CD19⁺ B cells mediate humoral immunity, and their excessive activation can produce a large amount of specific antibodies and may form immune complex deposits in the basement membrane of pleural capillaries; CD56⁺ NK cells, as an important component of innate immunity, their abnormal proportion can affect the release of inflammatory factors (11). Once the dynamic balance among these subgroups is disrupted, it can participate in the formation process of pleural effusion by altering the cytokine profile (such as TNF-α, IL-6) and damaging the integrity of the endothelial barrier (12). Previous studies have shown that the proportions of CD3⁺ T and CD4⁺ T cells in the peripheral blood of children with mycoplasma pneumonia can decrease as the disease progresses, while the changes in CD8⁺ T cells are inconsistent, and the changes in CD19⁺ B cells and CD56⁺ NK cells are also controversial (11, 13). However, these studies mostly focus on children with mycoplasma pneumonia overall, and there is insufficient evidence for the characteristic changes in lymphocyte subtypes of this specific subgroup with pleural effusion. There is also a lack of systematic comparative analysis. Immune abnormalities induced by MPP may promote pleural effusion by modulating cytokine release, impairing pathogen clearance, and damaging mucosal barrier integrity (14). Therefore, it is necessary to clarify whether there were any unique shifts in lymphocyte subpopulations in children with mycoplasma pneumonia complicated by pleural effusion, and to investigate the relationship between this shift and the occurrence of pleural effusion. This is an area that needs to be urgently filled in current research.

Accordingly, this retrospective study aimed to analyze the pathogenic characteristics, lymphocyte subset features, and risk factors associated with pleural effusion in children with MPP, to support early clinical diagnosis and treatment. All 104 children included in this study were confirmed cases of MPP through pathogen detection. Both groups had MPP as the common underlying disease, with the only difference being whether they had pleural effusion or not. The design of this study was expected to help eliminate the confounding influence of non-MPP causes of pleural effusion.

## 2 Materials and methods

### 2.1 Study objects

This study retrospectively selected 104 children diagnosed with mycoplasma pneumonia in the pediatric department of our hospital from February 2023 to February 2025 as the research subjects. Inclusion criteria: (1) Met the diagnostic criteria for MPP (15), and has been confirmed with mycoplasma pneumoniae infection through pathogen testing; (2) Presented with clinical features including poor feeding, dehydration, altered consciousness, or multi-lobe pulmonary involvement; (3) Pleural effusion was confirmed by chest ultrasonography; (4) Age between 1 and 13 years. Exclusion criteria: (1) patients with congenital anomalies of major organs; (2) patients complicated with pulmonary disorders including malignancy or tuberculosis; (3) patients who used glucocorticoids or immunomodulatory agents before the study; (4) patients with pleural effusion due to tumour, trauma, autoimmune disease, or other non-infectious aetiologies. Pleural effusion was diagnosed using chest ultrasonography, independently evaluated by two senior paediatric or radiology specialists. Diagnostic criteria were defined as follows (16): anechoic or hypoechoic intrapleural fluid collection measuring ≥10 mm in depth, or fluid <10 mm associated with clinically significant symptoms (dyspnoea, dullness to percussion, reduced breath sounds) requiring intervention. All imaging records were stored and re-evaluated to ensure accurate grouping.

### 2.2 Pathogenic analysis

Clinical samples of the patients were collected for pathogen detection. According to the patients' conditions and operational feasibility, the sample types were classified into the following three categories:

Airway secretions (including nasopharyngeal swabs and sputum): Applicable to all included patients, it is the preferred sample for collection. This sample is used for bacterial culture, mycoplasma pneumoniae culture, virus isolation, and real-time fluorescent quantitative PCR detection of mycoplasma pneumoniae and respiratory viruses (adenovirus, respiratory syncytial virus, influenza virus A/B types).

Bronchoalveolar lavage fluid: Collected only when bronchoscopy is routinely performed in clinical practice. The detection items of this sample are the same as those of airway secretions, including bacterial culture, Mycoplasma pneumoniae culture, virus isolation, and PCR detection.

Pleural effusion: Collected only for patients with pleural effusion during therapeutic puncture. This sample undergoes bacterial culture, virus culture, bacterial smear, and fungal culture, and is tested using real-time fluorescent quantitative PCR for Mycoplasma pneumoniae and viral nucleic acid.

All samples were incubated at 35 °C in a 5% CO_2_ incubator. Subsequent identification was carried out based on the type of pathogen: Bacterial identification was performed using the matrix-assisted laser desorption ionization time-of-flight mass spectrometry system (Autoflex Speed, Bruker, Germany) for species identification; Virus detection was performed using real-time fluorescent quantitative PCR (LightCycler® 480 II, Roche, Switzerland; nucleic acid extraction reagents purchased from Shanghai Zhenjiang Biotechnology Co., Ltd.), with specific primers designed for common respiratory viruses, including adenovirus (hexon gene), respiratory syncytial virus (N gene), influenza virus A/B types (M gene), etc.; Mycoplasma pneumoniae detection was performed by PCR amplification of its specific gene fragment (such as P1 protein gene, 16S rRNA gene), and sequence verification was conducted.

The detection methods (culture in media, matrix-assisted laser desorption ionization time-of-flight mass spectrometry identification, real-time fluorescent quantitative PCR) and positive determination criteria for all samples were the same. The positive determination criteria were: Typical colonies appeared in bacterial culture and the score of matrix-assisted laser desorption ionization time-of-flight mass spectrometry identification was ≥2.0; For virus and mycoplasma pneumoniae PCR detection, the cycle threshold (Ct) was <35, and the amplification curve was typical S-shaped; If the Ct value was between 35 and 38, it was re-tested once, and the re-test remained within this range or showed a typical amplification curve, it was judged as weakly positive (17). When pathogen positivity was combined for statistics, any specimen from the same patient that detected the same pathogen was counted as positive (including weakly positive). All operations were carried out strictly in accordance with the standard microbiological procedures, and positive and negative controls were set up. Bacterial culture used standard strains (such as Streptococcus pneumoniae ATCC 49619, Staphylococcus aureus ATCC 25923, etc.) as positive controls, and sterile normal saline was used as the negative control; PCR detection used plasmids containing target gene fragments or inactivated pathogen nucleic acid as positive controls, and no-nuclease water was used as the template as the negative control.

### 2.3 Clinical data collection

Baseline characteristics and laboratory parameters were recorded, including age, gender, pulmonary rales, hepatosplenomegaly, and radiological lesion distribution. Laboratory tests included complete blood count [white blood cell count (WBC), platelet count (PLT), haemoglobin (Hb)], liver function [aspartate transaminase (AST), alanine transaminase (ALT), creatine kinase (CK)], and inflammatory markers (α1-AG, ALB, CRP, GAG). Fever duration was also documented. MEK-7222K fully automatic blood analyzer (Shanghai Jumu Medical Equipment Co., Ltd.) was used for blood routine testing. The biochemical index detection adopted a CM-800 fully automatic biochemical analyzer (Jidan Biotechnology Co., Ltd.). All tests strictly followed standardized operating procedures and indoor quality control to ensure data reliability.

### 2.4 Lymphocyte subset analysis

Peripheral venous blood (2 mL) was collected before treatment and anticoagulated with EDTA. Peripheral blood mononuclear cells were isolated using density gradient centrifugation, washed with phosphate-buffered saline, and stained with fluorescent-conjugated monoclonal antibodies: CD3-FITC, CD4-PE, CD8-APC, CD19-PerCP, CD56-PE-Cy7. After incubation in the dark for 30 min, hemolysin was added to remove red blood cells. The samples were analyzed using a BD FACSCanto II flow cytometer. FlowJo software was used to determine the proportions of CD3 ^+^ T, CD4 ^+^ T, CD8 ^+^ T, CD19 ^+^ B, and CD56 ^+^ NK cells. Appropriate isotype controls and quality assurance measures were applied throughout to ensure the accuracy of typing results. The operational procedure for detecting peripheral blood lymphocyte subsets by flow cytometry was carried out according to the previous study (18).

### 2.5 Statistical analysis

Statistical analysis was performed using SPSS 26.0. Normally measurement data were expressed as mean ± standard deviation and compared using independent-samples *t*-tests. Non-normally measurement data were presented as median (interquartile range) and analyzed using the Mann–Whitney *U* test. Enumeration data were reported as numbers and percentages and compared using the *χ*^2^ test. The variables with *P* < 0.05 in the single-factor analysis (including fever duration, α1-AG, ALB, ALT, CRP, GAG) were included in the multivariate Logistic regression model (forward stepwise method) to analyze the independent influencing factors of pleural effusion in mycoplasma pneumonia. Results were reported as odds ratios (OR) with 95% confidence intervals (CI). The predictive value of the relevant indicators was evaluated using the receiver operating characteristic (ROC) curve, and the area under the curve (AUC) was compared using the *Z*-test. A *P*-value <0.05 was considered statistically significant.

## 3 Results

### 3.1 Patient inclusion

**Figure 1:**
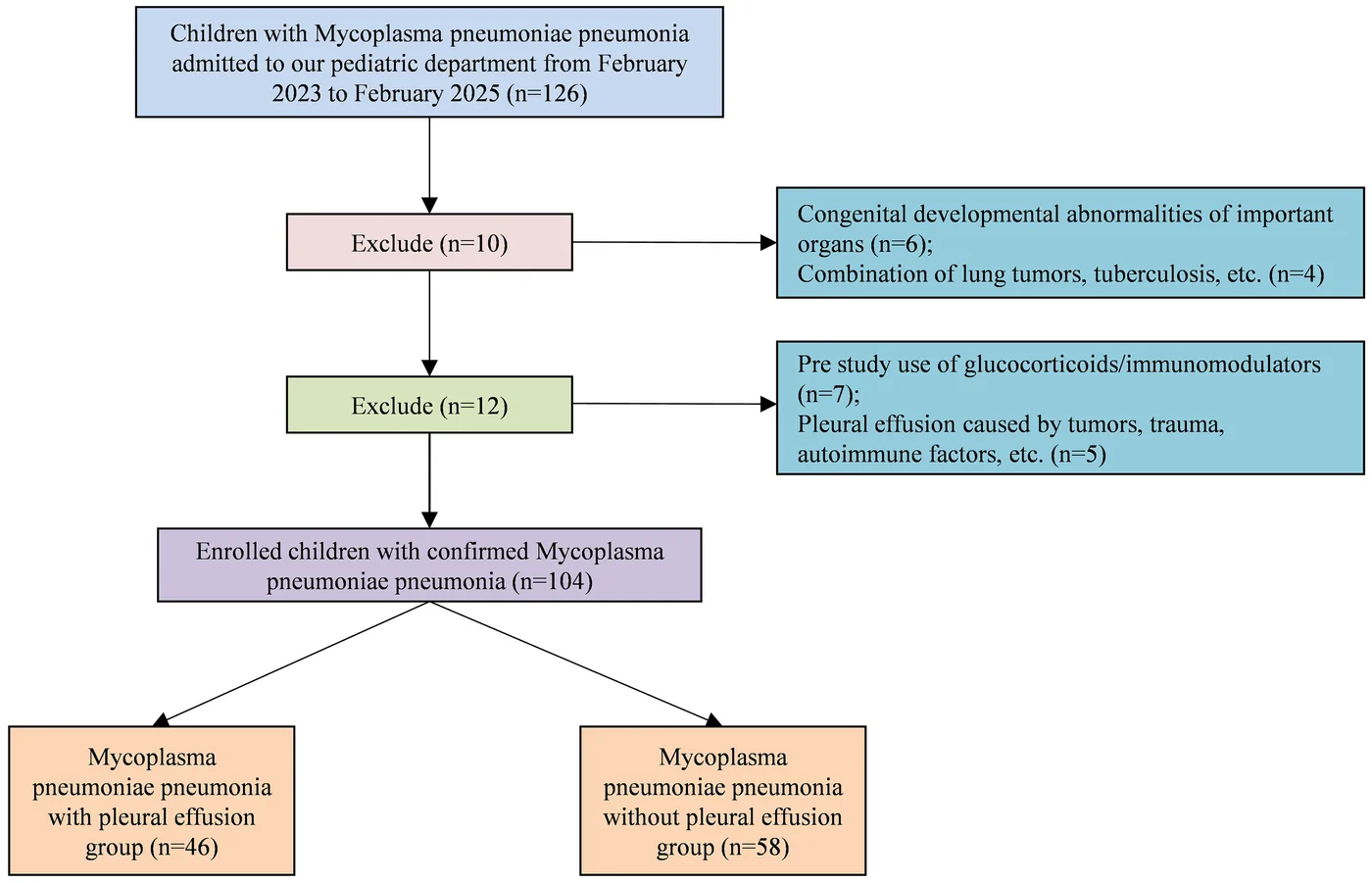
Patient inclusion process diagram.

From February 2023 to February 2025, a total of 104 children diagnosed with MPP in the pediatric department of our hospital were included in the retrospective study. The patients were divided into the MPP group with pleural effusion (*n* = 46) and the group without pleural effusion (*n* = 58). The patient inclusion process was shown in Figure 1.

### 3.2 Pathogenic characteristics of children with MPP and pleural effusion

Among 104 children with MPP, 46 were complicated with pleural effusion. A total of 59 pathogens were detected, including 19 strains of *M. pneumoniae* (32.20%), 14 bacterial strains (23.73%), and 26 viral strains (44.07%). Single infection accounted for 33.90%, and mixed infection accounted for 66.10% (Table 1).

**Table 1:** Pathogenic characteristics of children with MPP combined with pleural effusion [cases (%)].

Pathogen	No. of strains	Percentage (%)
Mycoplasma pneumoniae	19	32.20
Bacteria	14	23.73
Streptococcus pneumoniae	5	8.47
Staphylococcus aureus	2	3.39
Pseudomonas aeruginosa	1	1.69
Klebsiella pneumoniae	1	1.69
Others	5	8.47
Viruses	26	44.07
Adenovirus	15	25.42
Respiratory syncytial virus	6	10.17
Influenza virus	5	8.47
Total	59	100.00
Simple infection	20	33.90
Mixed infection	39	66.10

### 3.3 Comparison of pathogen distribution between children with Mycoplasma pneumonia combined with or without pleural effusion

No significant differences were observed between the MPP with pleural effusion group and the without pleural effusion group in infection type (single/mixed), main pathogen composition (*M. pneumoniae*/bacteria/viruses), or common mixed infection patterns (all *P* > 0.05, Table 2).

**Table 2:** Comparison of pathogen distribution between children with mycoplasma pneumonia combined with or without pleural effusion.

Pathogenic type	Group without pleural effusion associated with mycoplasma pneumoniae (*n* = 58)	MPP complicated with pleural effusion group (*n* = 46)	*χ*^2^ value	*P* value
Simple infection	24 (41.38)	12 (26.09)	2.651	0.104
Mixed infection	34 (58.62)	34 (73.91)		
Main pathogenic components			0.336	0.845
Mycoplasma pneumoniae	22 (37.93)	15 (32.61)		
Bacteria	11 (18.97)	9 (19.57)		
Viruses	25 (43.10)	22 (47.83)		
Common combinations of mixed infections			0.267	0.966
Virus + Mycoplasma pneumoniae	15 (25.86)	13 (28.26)		
Virus + bacteria	10 (17.24)	11 (23.91)		
Mycoplasma pneumoniae + bacteria	6 (10.34)	7 (15.22)		
Mixing of three pathogens	3 (5.17)	3(6.52)		

### 3.4 Comparison of clinical characteristics between the two groups

This study compared the inflammatory and biochemical indicators (such as α1-AG, CRP, GAG, etc.) between the two groups of children. The aim was to identify potential clinical characteristics related to the occurrence of pleural effusion, in order to provide candidate variables for subsequent multivariate regression analysis and predictive model construction. There were no significant differences between the two groups in age, gender, pulmonary rales, hepatosplenomegaly, lesion location, WBC, PLT, Hb, AST, or CK (all *P* > 0.05). Compared with the non-effusion group, the MPP with pleural effusion group had significantly longer fever duration and higher levels of α1-AG, ALT, CRP, and GAG, as well as significantly lower ALB (all *P* < 0.05, Table 3).

**Table 3:** Comparison of clinical characteristics between two groups [cases (%), (*x* ± *s*)].

Groups	Non-effusion group (*n* = 58)	MPP complicated with pleural effusion group (*n* = 46)	*t*/*χ*^2^	*P*
Age (years)	5.68 ± 1.27	5.19 ± 1.39	1.874	0.064
Gender			0.503	0.478
Male	30 (51.72)	27 (58.70)		
Woman	28 (48.28)	19 (41.30)		
Fever time (days)	3.82 ± 0.74	4.25 ± 1.25	2.183	0.031
Pulmonary rales	53 (94.38)	44 (95.65)	0.746	0.388
Hepatosplenomegaly	0 (0.00)	1 (2.17)	1.273	0.259
Lesion site			0.405	0.817
Left lung	21 (36.21)	14 (30.43)		
Right lung	19 (32.76)	17 (36.96)		
Bilateral	18 (31.03)	15 (32.61)		
WBC (×10^9^/L)	12.30 ± 2.84	13.17 ± 3.62	1.374	0.173
PLT (×10^9^/L)	254.26 ± 42.25	239.87 ± 56.79	1.482	0.142
Hb (g/L)	125.47 ± 7.35	128.30 ± 10.26	1.637	0.105
α1-AG (g/L)	1.52 ± 0.49	1.94 ± 0.74	3.470	0.001
ALB (U/L)	39.52 ± 3.11	37.85 ± 3.53	2.562	0.012
ALT (U/L)	15.93 ± 4.36	18.26 ± 5.24	2.475	0.015
AST (U/L)	25.84 ± 5.42	27.14 ± 6.37	1.124	0.264
CRP (mg/L)	10.41 ± 2.35	35.42 ± 7.70	23.425	<0.001
CK (U/L)	87.72 ± 25.34	93.64 ± 21.56	1.263	0.210
GAG (μg/mL)	34.27 ± 9.58	97.84 ± 14.85	26.415	<0.001

### 3.5 Comparison of peripheral blood lymphocyte subsets between the two groups

Compared with the non-effusion group, the MPP with pleural effusion group showed significantly lower percentages of CD3 ^+^ T, CD4 ^+^ T, and CD8 ^+^ T cells, and significantly higher percentages of CD19 ^+^ B and CD56 ^+^ NK cells (all *P* < 0.001, Table 4). The above results indicated that children with mycoplasma pneumonia accompanied by pleural effusion presented a state of cellular immune suppression characterized by a decrease in the proportion of T lymphocytes (CD3⁺, CD4⁺, CD8⁺), while the proportions of B lymphocytes (CD19⁺) and NK cells (CD56⁺) increased. This interplay of changes suggested that there was an imbalance between cellular immunity and humoral immunity in this group of children, and their immune system was in a state of dysfunction.

**Table 4:** Comparison of peripheral blood lymphocyte subsets between the two groups (mean ± SD, %).

Groups	Cases	CD3 + T	CD4 + T	CD8 + T	CD19 + B	CD56 + NK
Non-effusion group	58	55.90 ± 7.39	35.75 ± 6.53	15.42 ± 3.43	27.17 ± 8.23	14.13 ± 3.64
MPP complicated with pleural effusion group	46	35.83 ± 10.81	21.19 ± 5.06	11.69 ± 4.06	36.61 ± 10.33	21.47 ± 5.21
*t*		11.221	12.443	5.077	5.188	8.445
*P*		<0.001	<0.001	<0.001	<0.001	<0.001

### 3.6 Multivariate logistic regression analysis for pleural effusion in MPP

**Figure 2:**
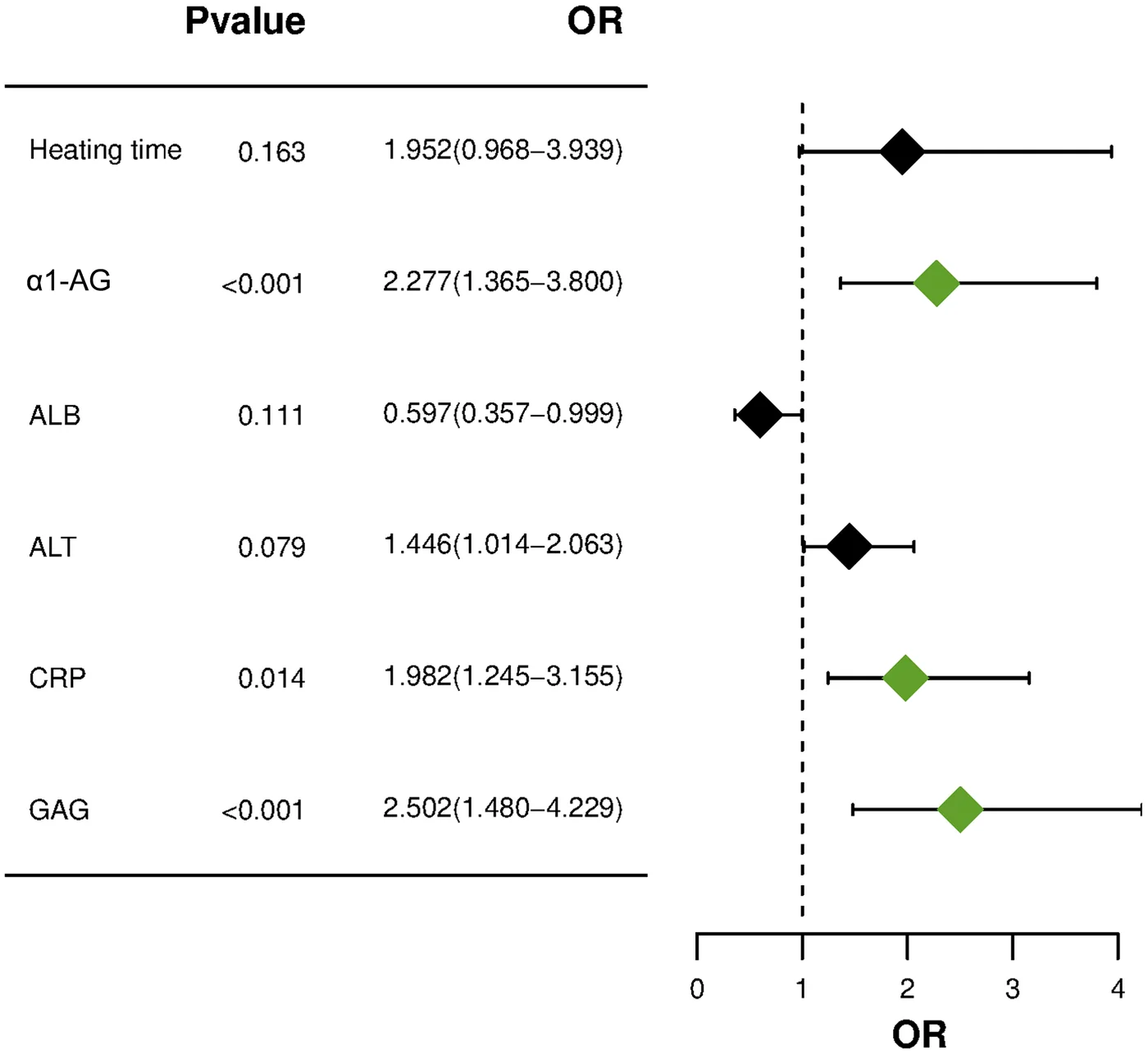
Forest plot of multivariate logistic regression analysis of Mycoplasma pneumonia complicated with pleural effusion.

Multivariate forward stepwise logistic regression was performed with the occurrence of pleural effusion as the dependent variable (yes = 1, no = 0), and variables with *P* < 0.1 in Univariate analysis as independent variables (including fever duration, α1-AG, ALB, ALT, CRP, GAG). The results showed that α1-AG, CRP, and GAG were independent risk factors for pleural effusion in children with MPP (all *P* < 0.05). Each 1 g/L increase in α1-AG increased the risk by 2.277-fold (95% CI: 1.365–3.800). Each 1 mg/L increase in CRP increased the risk by 1.982-fold (95% CI: 1.245–3.155). Each 1 μg/mL increase in GAG increased the risk by 2.502-fold (95% CI: 1.480–4.229). Fever duration, ALB, and ALT showed no independent significance after adjustment (all *P* > 0.05, Table 5 and Figure 2).

**Table 5:** Multivariate logistic regression analysis of MPP complicated with pleural effusion.

Indicator	*β*	SE	Wald	*P*	OR	95% CI
Fever time	0.669	0.358	3.492	0.163	1.952	0.968–3.939
α1-AG	0.823	0.261	9.943	<0.001	2.277	1.365–3.800
ALB	−0.516	0.262	3.879	0.111	0.597	0.357–0.998
ALT	0.369	0.181	4.156	0.079	1.446	1.014–2.063
CRP	0.684	0.237	8.329	0.014	1.982	1.245–3.155
GAG	0.917	0.268	11.708	<0.001	2.502	1.480–4.229

### 3.7 Predictive value of serum α1-AG, CRP, GAG and their combination

**Figure 3:**
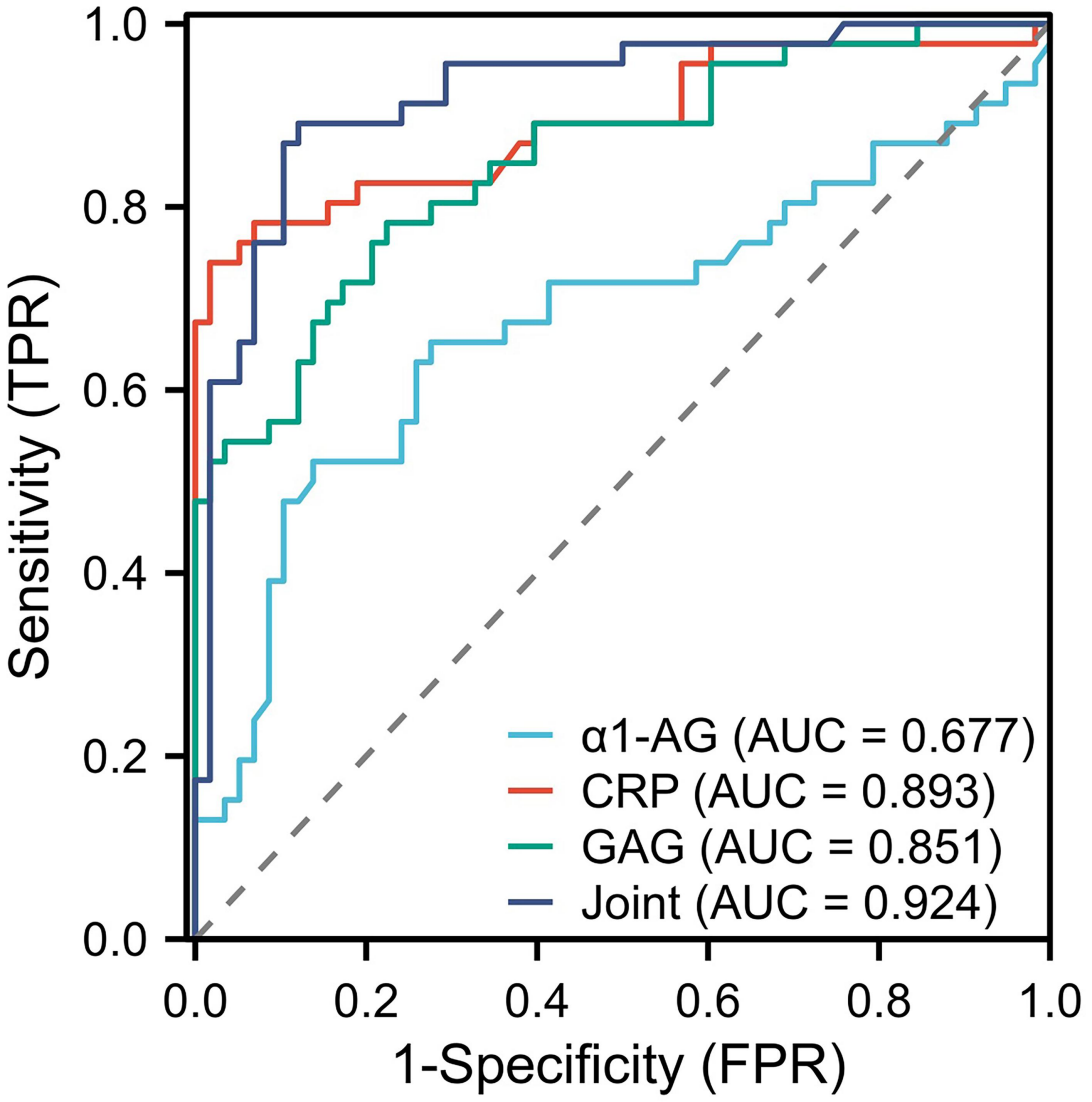
ROC curves of serum α1-AG, CRP, GAG and their combination for predicting pleural effusion in Mycoplasma pneumonia.

Receiver operating characteristic (ROC) curves were constructed to evaluate the predictive performance of these independent risk factors. As shown in Table 6 and Figure 3, CRP had the largest area under the curve (AUC = 0.893), with good discrimination (sensitivity 73.91%, specificity 98.28%). GAG (AUC = 0.851) and α1-AG (AUC = 0.677) also showed moderate predictive value. The combined model of α1-AG + CRP + GAG achieved an AUC of 0.924, with sensitivity 89.13% and specificity 87.93%, which was significantly better than any single marker.

**Table 6:** Predictive value of serum α1-AG, CRP, GAG and their combination for pleural effusion in MPP.

Indicators	AUC	95% CI	Sensitivity (%)	Specificity (%)	Cut-off value	Youden index
α1-AG	0.677	0.566–0.787	52.17	86.21	>1.75 g/L	0.384
CRP	0.893	0.824–0.961	73.91	98.28	>18.50 mg/L	0.722
GAG	0.851	0.777–0.925	78.26	77.59	>58.30 μg/mL	0.558
Combined detection	0.924	0.870–0.977	89.13	87.93	–	0.771

## 4 Discussion

Pleural effusion is one of the most common causes of worsening in patients with MPP. A previous study reported an incidence of 44.30% (39/88) (19), consistent with the 44.23% (46/104) observed in the present study, confirming a high prevalence of pleural effusion in paediatric MPP. Pathogenic analysis revealed that viruses were the most frequently detected pathogens, followed by *M.pneumoniae*, with mixed infections predominating. These findings highlight the importance of early pathogenic evaluation and antimicrobial susceptibility testing to guide individualised treatment and improve clinical outcomes. However, some studies have reported bacterial and *M.pneumoniae* as the primary pathogens (20), which may reflect differences in sample size, population characteristics, and study design.

Cellular and humoral immunity constitute the primary host defence systems, both of which can be impaired during *M.pneumoniae* infection, leading to immune imbalance. Lymphocyte subsets play critical roles in immune regulation: T lymphocytes mediate cellular immunity, B lymphocytes govern humoral immunity, and natural killer cells provide anti-infective and immunomodulatory functions (10). Li et al. (21) demonstrated that CD3 ^+^ T and CD4 ^+^ T cell proportions decline progressively with disease severity in MPP and correlate with the extent of pulmonary inflammation, suggesting that T-lymphocyte depletion contributes to disease progression. Wang et al. (22) reported that reduced CD4 ^+^ CXCR4 ^+^ T cells were associated with pleural effusion in children and provided experimental evidence supporting immunomodulatory therapy. In the present study, children with pleural effusion exhibited significantly lower CD3 ^+^ T, CD4 ^+^ T, and CD8 ^+^ T proportions and higher CD19 ^+^ B and CD56 ^+^ NK percentages, indicating profound immune dysregulation. Reduced cytotoxic CD8 ^+^ T cells may impair intracellular pathogen clearance, whereas elevated CD19 ^+^ B cells suggest excessive humoral activation, possibly related to immune complex formation or autoimmune responses. Although some studies reported decreased absolute CD56 ^+^ NK cells in severe MPP (23), the increased proportion observed in this study might reflect compensatory immune activation, which warrants further mechanistic exploration. The combined pattern of decreased T cells and increased B cells and NK cells in these children constitutes a unique immune dysregulation characteristic that distinguishes them from the non-exudate group (CD3⁺T: 55.90% vs. 35.83%; CD19⁺B: 27.17% vs. 36.61%; CD56⁺NK: 14.13% vs. 21.47%). From a functional perspective, the significantly decreased CD8⁺T cells in this group correspond to the high rate of viral co-infection mentioned earlier, suggesting that cellular immune suppression may weaken the host's ability to clear intracellular pathogens such as viruses, prolong the duration of local inflammatory stimulation, and provide an inflammatory basis for the continuous exudation of pleural capillaries (24). The reduction of CD4⁺T cells may further affect the Th1/Th2 balance and weaken the body's regulatory ability to limit the spread of inflammation (25). At the same time, the compensatory increase of CD19⁺B cells may promote the generation of MPP-specific antibodies and immune complexes, which deposit on the basement membrane of pleural capillaries and can increase vascular permeability through the activation of the complement cascade reaction (26). The increase in CD56⁺NK cells may be related to the compensatory mobilization under the inflammatory state of the body, but the inflammatory factors such as IFN-γ and TNF-α released by them can further exacerbate endothelial damage (13). From a pathophysiological perspective, the suppression of T cell-mediated anti-infection immunity may delay the clearance of pathogens and prolong the duration of local inflammation; the excessive activation of B cells may generate immune complexes and deposit on the basement membrane of pleural capillaries; the abnormal increase of NK cells may release excessive inflammatory factors, which can further increase the permeability of pleural capillaries (27, 28). It is reported that the absolute value of CD56⁺ NK cells in children with severe pneumonia mycoplasma infection was significantly lower than that in patients with mild infection (29). In this study, the proportion of CD56⁺ NK cells increased, which might be related to the compensatory activation of the immune system under disease conditions or changes in the function of specific subgroups. The specific significance of this needs to be further explored. Thus, children with MPP and pleural effusion exhibited a multiple immune imbalance pattern of “reduced cellular immunity, excessive activation of humoral immunity, and abnormal mobilization of innate immunity”. The coordinated action of these pathways jointly promoted pleural exudation and effusion formation.

Children with MPP and pleural effusion are at increased risk of long-term sequelae (30), highlighting the need to identify reliable risk factors. This study identified α1-AG, CRP, and GAG as independent risk factors for pleural effusion. α1-AG is an acute-phase protein closely linked to inflammation and immune function. Elevated levels of α1-AG have been associated with disease severity in pulmonary tuberculosis (31). CRP is a well-recognised acute-phase reactant that increases markedly during infection and inflammation. As an independent risk factor, CRP may reflect infection-induced immune activation and increased pleural capillary permeability, promoting pleural inflammation and fluid formation (32, 33). GAG is a key component of proteoglycans involved in cell signalling, inflammatory modulation, and immune regulation. During *M. pneumoniae* infection, excessive mucin secretion impairs tissue homeostasis, and GAG modulates inflammatory mediator activity to affect pleural fluid dynamics (34). Clinically, the risk factors for the occurrence of pleural effusion in *M.pneumonia* can be analyzed. Timely diagnosis and treatment can be carried out to reduce or avoid the occurrence of pleural effusion. However, due to limitations such as time and sample size, the analysis of influencing factors in this study might not be comprehensive. In the future, further evaluation of relevant factors could be conducted to more accurately assess the condition of pediatric patients.

ROC analysis indicated that CRP provided the best individual predictive performance (AUC = 0.893), followed by GAG (0.851) and α1-AG (0.677). The combined detection achieved an AUC of 0.924, with sensitivity 89.13% and specificity 87.93%, significantly superior to any single marker. These results were consistent with previous studies supporting the prognostic value of CRP in MPP (33) and demonstrated the advantage of combined multi-marker over single biomarker (35). The combined detection provides a clinically practical and high-performance tool for early risk stratification by integrating inflammatory activation (α1-AG), systemic inflammation (CRP), and immune regulation (GAG).

In summary, among the 104 children diagnosed with mycoplasma pneumonia in this group, respiratory pathogens were most commonly detected as viruses, and the rate of mixed infections was relatively high. Children with pleural effusion showed more significant immune dysfunction. The levels of serum α1-AG, CRP, and GAG were independent risk factors for mycoplasma pneumonia complicated with pleural effusion. The combined prediction model of these three indicators had a high diagnostic value (AUC = 0.924), suggesting that this model might provide a reference for clinical early warning. The three indicators covered by this model could be quickly obtained through peripheral blood routine tests, without the need for additional invasive operations. The detection cost was low, the turnaround time was short, and it was convenient to promote and use in various medical institutions. This helped clinical doctors to stratify patients with mycoplasma pneumonia in the early stage of the disease, timely identify high-risk groups for pleural effusion, and formulate individualized monitoring and intervention plans. Limitations include the retrospective single-centre design and relatively small sample size. Future multi-centre prospective studies with long-term follow-up are warranted to validate these findings and explore underlying immunopathogenic mechanisms.

## Data Availability

The original contributions presented in the study are included in the article/Supplementary Material, further inquiries can be directed to the corresponding author.
